# *In Vitro* Cell Density Determines the Sensitivity of Hepatocarcinoma Cells to Ascorbate

**DOI:** 10.3389/fonc.2022.843742

**Published:** 2022-05-23

**Authors:** Hsiu-Lung Fan, Shu-Ting Liu, Yung-Lung Chang, Yi-Lin Chiu, Shih-Ming Huang, Teng-Wei Chen

**Affiliations:** ^1^ Graduate Institute of Medical Sciences, National Defense Medical Center, Taipei, Taiwan; ^2^ Division of General Surgery, Department of Surgery, Tri-Service General Hospital, National Defense Medical Center, Taipei, Taiwan; ^3^ Department of Biochemistry, National Defense Medical Center, Taipei, Taiwan

**Keywords:** hepatocellular carcinoma, ascorbate, reactive oxygen species, tyrosine kinase inhibitors, cell density

## Abstract

Hepatocellular carcinoma (HCC) is the primary histological subtype of liver cancer, and its incidence rates increase with age. Recently, systemic therapies, such as immune checkpoint inhibitors, monoclonal antibodies, and tyrosine kinase inhibitors (TKIs), have been more beneficial than conventional therapies for treating HCC. Nonetheless, the prognosis of late-stage HCC remains dismal because of its high recurrence rates, even with substantial advances in current therapeutic strategies. A new treatment, such as a combination of current systemic therapies, is urgently required. Therefore, we adopted a repurposing strategy and tried to combine ascorbate with TKIs, including lenvatinib and regorafenib, in HepG2 and Hep3B cells. We investigated the potential functional impact of pharmacological concentrations of ascorbate on the cell-cycle profiles, mitochondrial membrane potential, oxidative response, synergistic effects of lenvatinib or regorafenib, and differential responsiveness between HepG2 and Hep3B cells. Our data suggest that the relative level of cell density is an important determinant for ascorbate cytotoxicity in HCC. Furthermore, the data also revealed that the cytotoxic effect of pharmacological concentrations of ascorbate might not be mediated *via* our proposed elevation of ROS generation. Ascorbate might be involved in redox homeostasis to enhance the efficacy of TKIs in HepG2 and Hep3B cells. The synergistic effects of ascorbate with TKIs (lenvatinib and regorafenib) support their potential as an adjuvant for HCC targeted TKI therapy. This research provides a cheap and new combinatory therapy for HCC treatment.

## Introduction

Liver cancer is the sixth most frequent cancer and the fourth-leading cause of death worldwide, with 841,000 new cases and 782,000 deaths in 2018, accounting for 7% of all cancers (1–3). Hepatocellular carcinoma (HCC) is the primary histological subtype of liver cancer, and its incidence rates, which peak at around 70 years old, are progressively increasing with age. HCC has a significant male preponderance. The incidence rate of men getting HCC is 2–2.5-fold higher than that of women (1). An already-identified underlying etiology, such as HIV infection, chronic hepatitis virus (HBV and HCV) infections, aflatoxin exposure, cigarette smoking, alcohol intake, and metabolic diseases, resulted in approximately 90% of HCCs. The prognosis of late-stage HCC remains dismal due to its high recurrence rates even with substantial advances in current therapeutic strategies. HCC has a poor 5-year survival rate, resulting from late diagnoses, resistance to anticancer therapies, and a high frequency of recurrences (2). Therefore, for the sake of developing new biomarkers for diagnoses and prognoses, as well as inventing effective treatments and drugs, elucidating the underlying tumorigenesis of HCC is important.

Owing to the significant progress of systemic therapies accompanied by a substantial increase in patients’ overall survival rates and 5-year quality of life, applying systemic therapies, such as monoclonal antibodies, immune checkpoint inhibitors (ICIs), and tyrosine kinase inhibitors (TKIs), to HCC patients has recently been more beneficial than traditional therapies (4–7). Sorafenib and lenvatinib are the most effective TKIs and first-line single-drug therapies. Cabozantinib, ramucirumab, and regorafenib have also been recognized to improve survival benefits in second-line therapies after first-line treatment with sorafenib. Many phase III trials have been conducted to investigate the effectiveness of combination therapy, for example. trials investigating the ICI combination of PD-1/PDL-1 (programmed death-1/programmed death ligand-1) axis inhibitors and CTLA4 (cytotoxic T-lymphocyte-associated protein 4) inhibitors or the combination of ICIs and TKIs are ongoing (6). The management of HCC at all stages is expected to be changed by the results of these trials. However, developing resistance is a primary problem for the antitumor effect of first-line and second-line TKIs in HCC (8, 9).

Screening for repurposed drugs is a potential strategy for identifying new cancer therapies (10). It has been hypothesized that the steady-state levels of reactive oxidative species (ROS) stress in cancer cells are higher than in normal cells owing to a defective oxidative metabolism and raised labile iron pool (11, 12). Hence, redox homeostasis plays a significant role in cancer therapy. Recently, l-ascorbic acid (l-AA) at pharmacologic concentrations (>100 μM) achieved by intravenous administration was redefined as a possible anti-cancer drug acting *via* inducing hydrogen peroxide formation (13–17), in contrast to physiological concentrations that neutralize free radicals/ROS. However, the reason that l-AA kills some tumor cells *via* hydrogen peroxide-related mechanisms but has little effect on normal cells is still under investigation (11, 12). In addition to ROS generation, ascorbate can also serve as a cofactor of hydroxylases to keep the active site Fe^2+^ in its reduced state (18). Recent advances have shown that the ascorbate-induced cell death of cancer cells involves DNA double-strand breaks and ATP depletion (19, 20). Ferroptosis, necroptosis, and autophagy are involved in caspase-independent pharmacological ascorbate-induced cell death (21).

For *in vitro* toxicity studies, two of the most utilized liver cancer cellular models are the HepG2 and Hep3B cancer cell lines (22, 23). HepG2 has wild-type p53 and is HBV-negative and nontumorigenic, whereas Hep3B is p53-deficient, HBV-positive, and tumorigenic. Many studies have claimed that the differences between HepG2 and Hep3B cell lines cannot merely be determined by p53 or HBx. Moreover, there also exist several differences between HepG2 and Hep3B cell lines, such as differences in gene expression, drug responses, and associated signaling pathways, as described in the literature (24–26). These diverse differences have constantly presented obstacles and led to confusion for many researchers attempting to analyze and interpret the experimental data.

In this study, we investigated the cytotoxic effect of ascorbate (sodium ascorbate or l-AA) on the HepG2 cell line whether mediated through the modulation of ROS status. We further examined the potential functional impact of pharmacological concentrations of ascorbate on the cell-cycle profiles, mitochondrial membrane potential, oxidative response, the synergistic effects of vascular endothelial growth factor receptor (VEGFR)-targeted TKIs, first-line lenvatinib, and second-line regorafenib, and the differential responsiveness between HepG2 and Hep3B cells. Our findings may provide a new direction for combinatory HCC therapy using ascorbate (sodium ascorbate or l-AA), focusing on the management of cellular redox homeostasis.

## Materials And Methods

### GSVA Scoring and Survival Analysis

Sorafenib FDA, lenvatinib FDA, regorafenib FDA, and cabozantinib FDA downloaded from DsigDB (27), and the gene sets associated with ROS downloaded from Gene Ontology (28). About the prognosis of HCC patients, TCGA LIHC whole gene expression and curated survival status were downloaded from the UCSC XENA website (29). In GSVA analysis (in R 4.1.1), kfcd was set to “Poisson”, and four TKI gene sets and ROS-related GO gene sets were input for scoring (30). TKI GSVA scores and survival status were entered into the “Evaluate Cutpoints” package (in R 4.1.1) to find the best cut-off point (31), and Kaplan-Meier plots were plotted with GraphPAD software. Log-rank test was used for statistically significant analysis, and p < 0.05 was considered a significant difference. The Heatmap showing GSVA scores is drawn with the Morpheus web tool. Violin plots were plotted with GraphPAD software, and Welch’s t-test was used for the analysis of statistical significance. ***: p < 0.001. For detailed software settings, please refer to the previous article (32, 33).

### Cell Culture and Reagents

HepG2 and Hep3B HCC cell lines were obtained from the American Type Culture Collection (ATCC; Manassas, VA). HepG2 and Hep3B cells were cultivated in Dulbecco’s modified Eagle’s medium (DMEM) supplemented with 10% fetal bovine serum (FBS) and 1% penicillin-streptomycin (Thermo Fisher Scientific, Waltham, MA). Cell lines were regularly tested for mycoplasma infections using Mycoplasma Detection Kit (*In vivo* Gen, San Diego, CA) as well as regularly changed with thawed stocking cell lines. l-AA, sodium ascorbate, 2′,7-dichlorofluorescein diacetate (DCFH-DA), lenvatinib, propidium iodide (PI), regorafenib, and thiazolyl blue tetrazolium bromide (MTT) were purchased from Sigma Aldrich (St. Louis, MO).

### Cell Metabolic Activity Analysis

Cells were plated into 24-well culture plates and incubated for 1 day, after which fresh DMEM containing the indicated drugs was added to each well. The cells were then incubated with these treatments for the indicated periods. The interaction between l-AA and MTT assay has already been demonstrated (34). Accordingly, to avoid the interference of l-AA with MTT, the l-AA-containing medium was removed before the addition of MTT solution (0.5 mg/mL in phosphate-buffered saline, PBS) to each well. The cells were then incubated with MTT solution for 1 h at 37°C. After adding dimethyl sulfoxide (DMSO; 200 μL), the absorbances at 570 nm and 650 nm were measured using an ELISA plate reader (Multiskan EX, Thermo, MA). The relative metabolic activity was calculated based on the absorbance ratio between cells cultured with the indicated drugs and the untreated controls, which were assigned a value of 100. The combination index (CI) was calculated utilizing CalcuSyn (Biosoft, Cambridge, UK) to generate the isobolograms. Typically, a CI value <1 denotes a synergistic combination effect, and a CI value >1 denotes an antagonistic combination effect (35).

### Fluorescence-Activated Cell Sorting (FACS), Cell-Cycle Profiling, and ROS and Mitochondrial Membrane Potential Analyses

Cell-cycle profiles were measured according to cellular DNA content using FACS. Cells were fixed in 70% ice-cold ethanol, stored at −30°C overnight, washed two times with ice-cold PBS supplemented with 1% FBS, and then stained with PI solution (5 μg/mL PI in PBS, 0.5% Triton X-100, and 0.5 μg/mL RNase A) for 30 min at 37°C in the dark.

The intracellular ROS levels were determined using the fluorescent marker DCFH-DA. Cells were exposed to various concentrations of ascorbate for 24 h, stained with DCFH-DA (10 μM) for 40 min at 37°C, and then harvested. After washing the cells once with PBS, fluorescence was analyzed on channel FL-1 of the FACSCalibur flow cytometer using Cell Quest Pro software (BD Biosciences, Franklin Lakes, NJ). The cell volume gating strategy involved forward scatter height (FSC-H) and side scatter height (SSC-H), and the median fluorescence intensity of the vehicle was used as the starting point for M2 gating.

Mitochondrial depolarization was measured as a function of a decrease in the red/green fluorescence intensity ratio. All dead and viable cells were harvested, washed with PBS, and incubated with 1× binding buffer containing the MMP-sensitive fluorescent dye JC-1 for 30 min at 37°C in the dark. After washing the cells once with PBS, JC-1 fluorescence was analyzed on channels FL-1 and FL-2 of the FACSCalibur flow cytometer using Cell Quest Pro software (BD Biosciences, Franklin Lakes, NJ) to detect monomer (green fluorescence) and aggregate (red fluorescence) forms of the dye, respectively. The cell volume gating strategy involved FSC-H and SSC-H, and the median fluorescence intensity of the vehicle was used as the starting point for M2 gating.

### Western Blotting

HepG2 and Hep3B cells were lysed in radioimmunoprecipitation assay buffer (100 mM Tris-HCl (pH 8.0), 150 mM NaCl, 0.1% SDS, and 1% Triton X-100) at 4°C. Proteins in the resultant lysates were separated by sodium dodecyl sulfate-polyacrylamide gel electrophoresis and analyzed by immunoblotting with antibodies against α-actinin (ACTN)(H-2), fatty-acid synthase (FAS)(A-5), Nrf2 (A-10), p53 (DO-1), p62 (D-3) (Santa Cruz Biotechnology, Santa Cruz, CA), cleaved poly-ADP-ribose polymerase (PARP) (9546) (Cell Signaling, Danvers, MA), cyclin D1 (ab134175), γH2A.x (ab81299), p21 (ab109520) (Abcam, Cambridge, UK), and HO-1 (heme oxygenase-1) (ADI-SPA-895-F) (Enzo Life Sciences, Farmingdale, NY).

### Statistical Analysis

Values were expressed as the mean ± SD of at least three independent experiments. All comparisons between groups (vehicle and drug) were conducted using Student’s *t*-tests. Statistical significance was set at *p* < 0.05.

## Results

### The Relationship Between TKIs and ROS in Human Liver Cancer Cells

**Figure 1:**
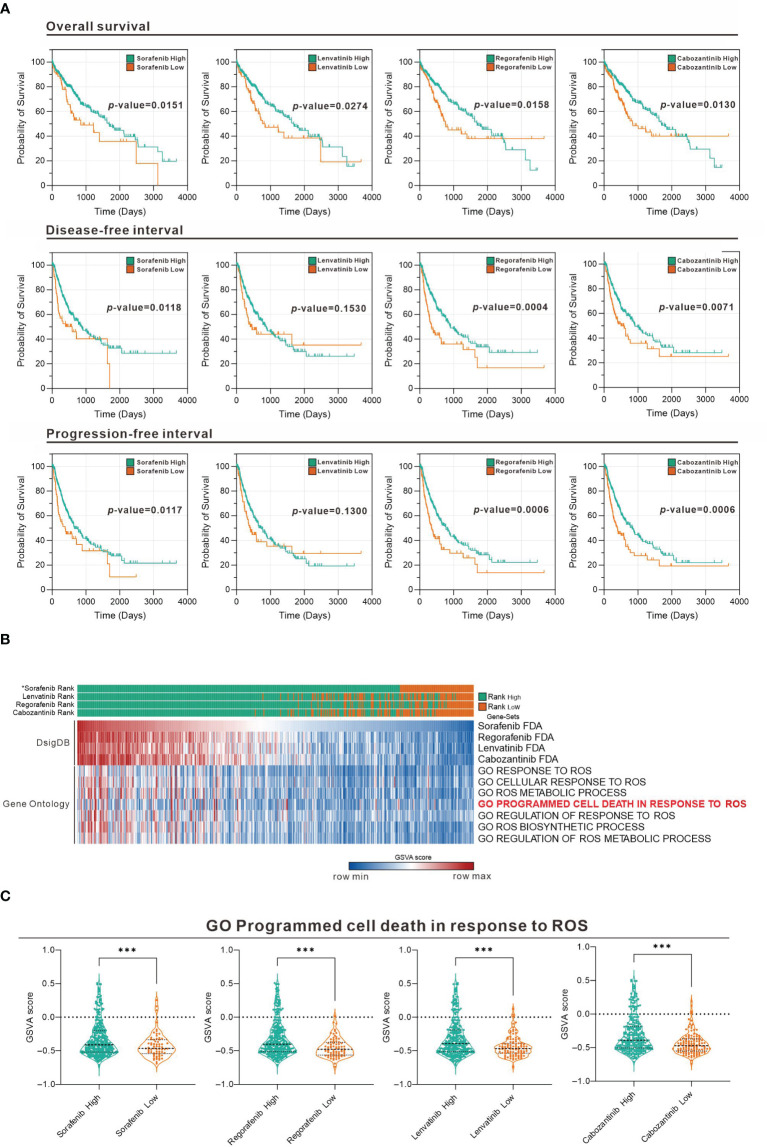
Association between TKI and ROS-responsive gene sets assessed in the TCGA LIHC database. **(A)** Overall survival, disease-free interval, and progression-free interval analysis for high and low GSVA scoring for the four drugs (sorafenib, lenvatinib, regorafenib, and cabozantinib). **(B)** Heatmap showing the distribution of the four-drug GSVA high and low and ROS-associated gene sets in TCGA LIHC patients. *: sorted by sorafenib GSVA scores from highest to lowest (sorafenib, lenvatinib, regorafenib, and cabozantinib). **(C)** Violin plot showing the difference in GSVA scores of GO programmed cell death in response to ROS in the high and low GSVA subgroups of the four drugs. ***p < 0.001 (Welch's t-test).

To evaluate the association of sorafenib and related TKI with ROS response in HCC, we used Gene Set Variation Analysis (GSVA) strategy in combination with four gene sets collected from D1: Food and Drug Administration (FDA) Approved Drug Gene Sets in Drug SIGnatures DataBase (DsigDB) for GSVA scoring. The Evaluate Cutpoints package in the R environment was used to find the best cut-off point for the survival rate of each gene set in The Cancer Genome Atlas (TCGA) Liver Hepatocellular Carcinoma (LIHC) database. **Figure 1A** shows that among the four-drug (sorafenib, lenvatinib, regorafenib, and cabozantinib) gene sets in GSVA scoring, those with higher scores generally had a better prognosis, while those with lower scores generally had a worse prognosis. Considering that the gene set represents genes with increased expression after drug treatment, the poor prognosis of those with lower GSVA scoring implies that lower expression of genes associated with TKI drug treatment in tumors may be associated with poor drug response. Further analysis of the distribution of GSVA scoring and ROS response GSVA scores among the four drugs in the TCGA LIHC patient sample showed that the low GSVA scoring was concentrated in specific patient groups (**Figure 1B**). Similarly, the GSVA scoring of the ROS-associated Gene Ontology (GO) gene-set was also low in this cohort of patients, suggesting that the effect of TKI drugs on tumor suppression by increasing ROS was limited in this cohort. Take “GO Programmed cell death in response to ROS” as an example, we found that all patients with low GSVA scoring of the four drugs had a significantly lower distribution of this gene set (**Figure 1C**). This indicates that the expression of genes related to ROS-induced apoptosis by the four TKI is low in this group of patients, which may be related to the reduced effect of TKI.

### The Cytotoxicity of Ascorbate in Human Liver Cancer Cells

**Figure 2:**
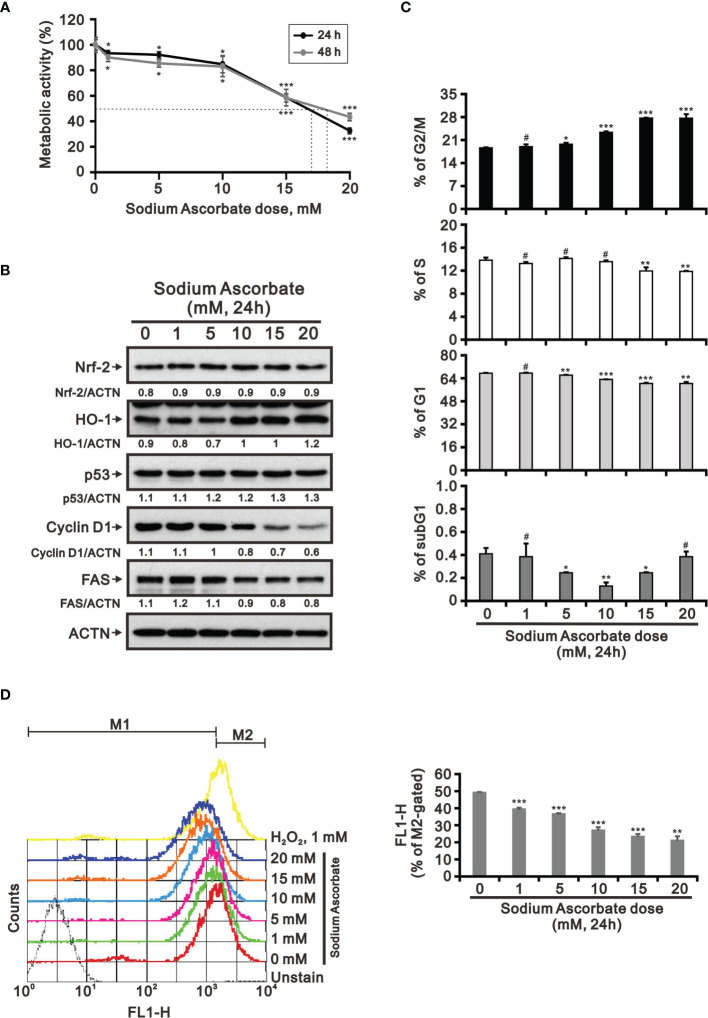
Effects of sodium ascorbate on cell metabolic activity in HepG2 cells. **(A)** HepG2 cells were treated for 24 h or 48 h with the indicated concentrations of sodium ascorbate. Cell metabolic activity was measured using the MTT assay. HepG2 cells were treated with the indicated concentrations of sodium ascorbate for 24 h, after which their cell lysates were subjected to **(B)** Western blot analysis using antibodies against the indicated proteins (ACTN was the protein loading control), **(C)** cell-cycle profiling using flow cytometry analysis, and **(D)** ROS status determination using 10 μM DCFH-DA. The cell volume gating strategy involved FSC-H and SSC-H, and the median fluorescence intensity of the vehicle was used as the starting point for M2 gating. Protein bands were quantified through pixel density scanning and evaluated using ImageJ, version 1.44a (http://imagej.nih.gov/ij/). We show one representative result. Bars depict the mean ± SD of three independent experiments. ^#^ *p* > 0.05, *p < 0.05, **p < 0.01, and ***p < 0.001 compared with the vehicle (Student’s t-tests).

Previous studies including ours on human cervical cancer cells have suggested that pharmacologic concentrations of ascorbate (l-AA or sodium ascorbate) may elevate ROS generation and result in cancer cell death (13, 17, 36–38). Based on the conclusion of **Figure 1**, the functional role of ROS generation by ascorbate might be a candidate for HCC treatment. Hence, we applied various concentrations, including pharmacologic concentrations, to HepG2 cells. Our MTT analysis for cell metabolic activity showed that sodium ascorbate treatment for either 24 h or 48 h could cause 50% HepG2 cell death at 15 mM (**Figure 2A**). We further analyzed the effects of sodium ascorbate on cell-cycle progression using Western blotting analysis and on cell-cycle profile using flow cytometry. According to the Western blotting analysis, cyclin D1 and fatty-acid synthase (FAS) were downregulated by sodium ascorbate, whereas the quantitative values of Nrf2 remained constant and the quantitative values of HO-1 and p53 were increased by a dose-dependent manner (**Figure 2B**). According to the cell-cycle profile, the population in the G2/M phase was significantly increased by sodium ascorbate, whereas the populations in the G1 and S phases were decreased (**Figure 2C**). No apparent impact on the subG1 phase of sodium ascorbate was observed, suggesting that the induction of cell death by sodium ascorbate might not be mediated *via* cellular apoptosis. Unexpectedly, we found that the ROS levels did not increase accordingly, indicating that the cell death caused by sodium ascorbate might not be caused by the induction of ROS generation (**Figure 2D**). The declining trend of ROS by sodium ascorbate was a dose-dependent manner.

### The Effect of HepG2 Cell Density on the Cytotoxicity of Ascorbate

**Figure 3:**
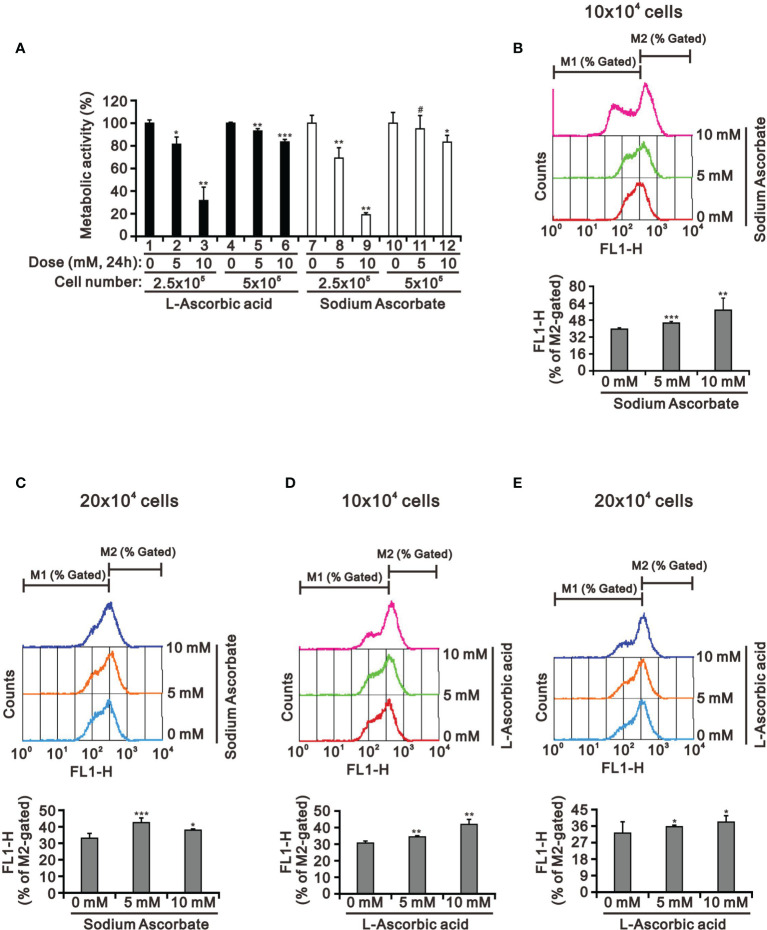
The cell density effect on cell metabolic activity and mitochondrial membrane potential in HepG2 cells. The two indicated cell densities of HepG2 cells were treated with 5 mM or 10 mM l-AA (or sodium ascorbate) for 24 h, after which their cell lysates were investigated for their **(A)** cell metabolic activity using the MTT assay and **(B–E)** mitochondrial membrane potential using a flow cytometer. The cell volume gating strategy involved FSC-H and SSC-H, and the median fluorescence intensity of the vehicle was used as the starting point for M2 gating. We show one representative result. Bars depict the mean ± SD of three independent experiments. ^#^ *p* > 0.05, *p < 0.05, **p < 0.01, and ***p < 0.001 compared with the vehicle (Student’s t-tests).

Our previous study had tested whether the weak acidity of l-AA was responsible for its observed effects by assessing the effects of sodium ascorbate salt and acetic acid in HeLa cells (17). Various sensitivities of ascorbate were previously identified as a function of cell density (39, 40). Therefore, we looked into the potential cell death-inducing effect of l-AA and sodium ascorbate using cell densities ranging from 2.5 × 10^5^ to 5 × 10^5^. Cell viability analysis using the MTT assay showed that 10 mM l-AA and sodium ascorbate had dramatically suppressive effects on HepG2 cell growth at lower cell density (**Figure 3A**). To be more specific, the lower the cell density was, the higher the possibility that ascorbate could enter into cells. Therefore, it might result in the accumulation of a higher amount of ascorbate inside HepG2 cells, leading to inducing a more significant cytotoxic effect. The lack of an apparent difference between l-AA and sodium ascorbate suggests the absence of a functional role of weak acidity of l-AA concerning cytotoxicity in HepG2 cells.

There was no apparent cause for the suppression of HepG2 cell growth revealed by the cell-cycle profile and cellular apoptosis. Hence, we checked the impact of l-AA and sodium ascorbate on mitochondria membrane potential at different cell densities using JC-1 dye. Our JC-1 data showed that l-AA and sodium ascorbate potentially disrupted mitochondrial membrane potential in a dose-dependent manner at lower cell density (**Figures 3B–E**).

### The Combinatory Effect of Ascorbate With TKIs in HepG2 Cells

**Figure 4:**
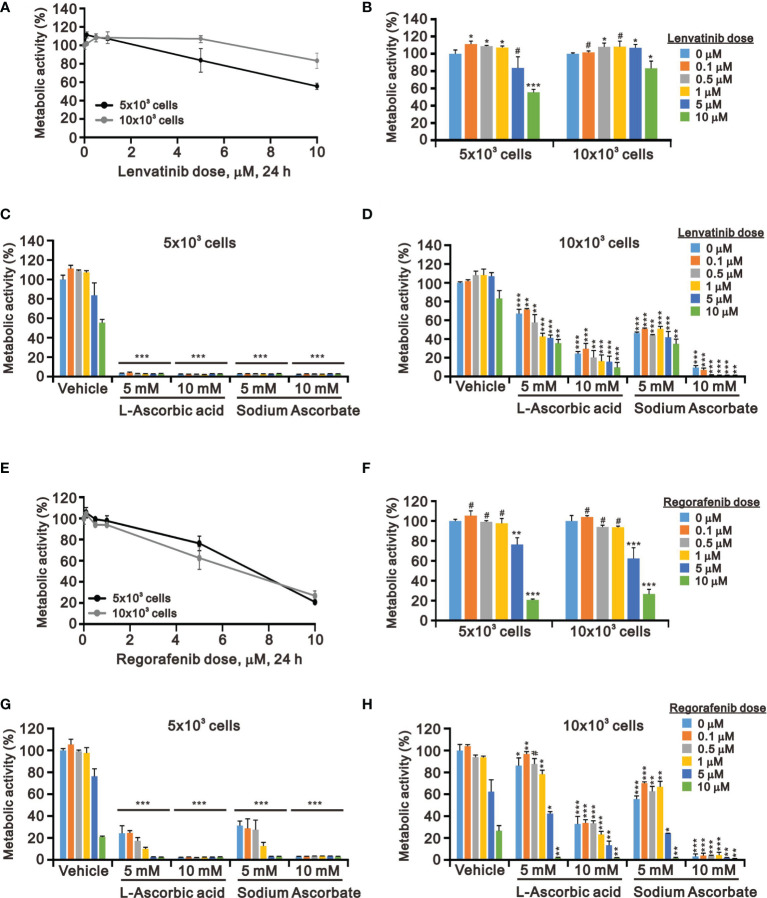
Effects of ascorbate combined with TKIs on cell viability in HepG2 cells. HepG2 cells (5 × 10^3^ and 1 × 10^4^) were treated for 24 h with the indicated concentrations of **(A–D)** lenvatinib or **(E–H)** regorafenib in the absence or presence of 5- or 10-mM l-AA or sodium ascorbate. Cell metabolic activity was measured using the MTT assay. We show one representative result. Bars depict the mean ± SD of three independent experiments. ^#^ *p* > 0.05, *p < 0.05, **p < 0.01, and ***p < 0.001 compared with the vehicle (Student’s t-tests).

Targeted TKI therapy is currently used for HCC treatment (6, 41, 42). The development of resistance is a primary problem for the antitumor effect of first-line and second-line TKIs in HCC (8, 9). Therefore, it is an urgent issue to reduce the recurrence rates by replacing currently targeted monotherapies with combinatory therapies. Lenvatinib is a multiple TKI of the VEGFR1–3 kinases and regorafenib demonstrates an antiangiogenic effect owing to its dual VEGFR2/TIE2 tyrosine kinase inhibition (43, 44). At pharmacologic concentrations, 10 mM l-AA or sodium ascorbate had more suppressive effects on cell metabolic activity than lenvatinib and regorafenib (except 10 μM) in HepG2 cells independent of cell density (**Figure 4**). HepG2 cells were sensitive to lenvatinib treatment at a lower cell density in a dose-dependent manner (**Figures 4A, B**). The growth of HepG2 cells was almost suppressed by l-AA or sodium ascorbate at a lower (5 × 10^3^) cell density (**Figure 4C**). We observed the combinatory cytotoxic efficiencies of lenvatinib and l-AA or sodium ascorbate at a 10 × 10^3^ cell density (**Figure 4D**). Compared with lenvatinib, HepG2 cells were more sensitive to regorafenib in a cell density-independent manner (**Figures 4E, F**). The combinatory cytotoxic efficiencies of regorafenib with l-AA or sodium ascorbate were dependent on the concentration of ascorbate and the cell density (**Figures 4G, H**).

**Figure 5:**
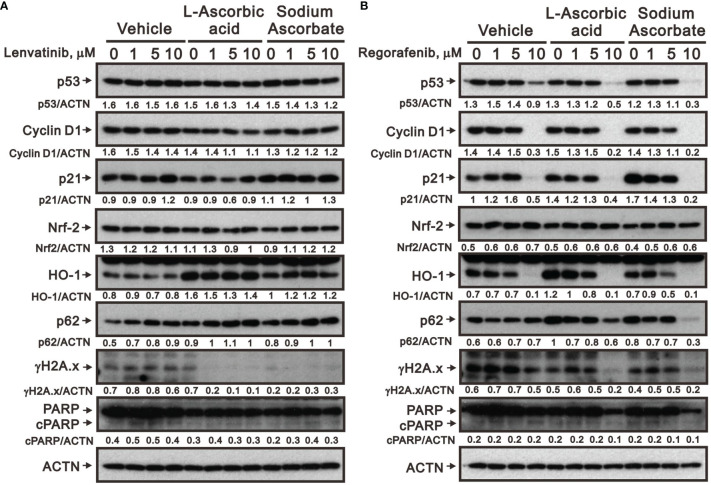
Effects of ascorbate combined with TKIs on specific protein expressions in HepG2 cells. HepG2 cells (4 × 10^5^) were treated for 24 h with the indicated concentrations of **(A)** lenvatinib or **(B)** regorafenib in the absence or presence of 5-mM l-AA or sodium ascorbate. Cell lysates were subjected to Western blot analysis using antibodies against the indicated proteins. ACTN was the protein loading control. Protein bands were quantified through pixel density scanning and evaluated using ImageJ, version 1.44a (http://imagej.nih.gov/ij/). We show one representative result.

We further elucidated the potential cytotoxicity mechanism of combination therapy of ascorbate with TKIs. We examined proteins related to cell-cycle progression, ROS response, DNA damage, and apoptosis using Western blotting analysis. We observed that lenvatinib alone induced the expression of cell-cycle progression-related proteins (p53 and p21), a ROS response protein (p62), a DNA damage biomarker (γH2A.x), and an apoptosis biomarker (cleaved PARP), while it downregulated the expression of an ROS response protein (HO-1) and a cell-cycle progression-related protein (cyclin D1) (**Figure 5A**). Lenvatinib combined with 5 mM l-AA or sodium ascorbate downregulated the expression of a cell-cycle progression-regulated protein (cyclin D1), a DNA damage biomarker (γH2A.x), and an apoptosis biomarker (cleaved PARP), whereas it elevated the quantitative values of a cell-cycle progression-related protein (p21) and ROS response proteins (HO-1 and p62). Regorafenib alone decreased the quantitative values of p53, cyclin D1, p21, and HO-1, while it elevated the quantitative values of p62. Regorafenib combined with 10 mM l-AA or sodium ascorbate downregulated the expression of cell-cycle progression-related proteins (p53, cyclin D1, and p21), ROS response proteins (Nrf2 and p62), a DNA damage biomarker (γH2A.x), and an apoptosis biomarker (cleaved PARP) (**Figure 5B**). γH2A.x was the only protein consistent change (decreased) in the combinations of lenvatinib or regorafenib with higher concentrations of ascorbate.

### The Cytotoxicity of Sodium Ascorbate in Hep3B Cells

**Figure 6:**
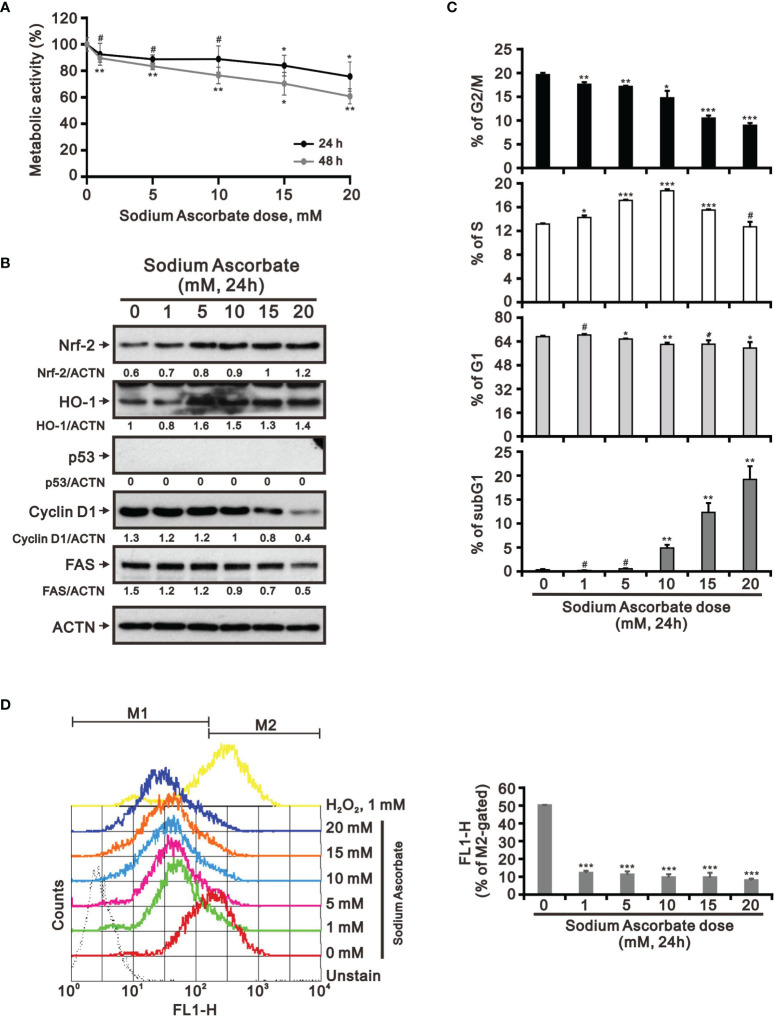
Effects of sodium ascorbate on cell metabolic activity in Hep3B cells. **(A)** Hep3B cells were treated for 24 h or 48 h with the indicated concentrations of sodium ascorbate. Cell metabolic activity was measured using the MTT assay. Hep3B cells were treated with the indicated concentrations of sodium ascorbate for 24 h, after which their cell lysates were subjected to **(B)** Western blot analysis using antibodies against the indicated proteins (ACTN was the protein loading control), **(C)** cell-cycle profiling using flow cytometry analysis, and **(D)** ROS status determination using 10 μM DCFH-DA. The cell volume gating strategy involved FSC-H and SSC-H, and the median fluorescence intensity of the vehicle was used as the starting point for M2 gating. Protein bands were quantified through pixel density scanning and evaluated using ImageJ, version 1.44a (http://imagej.nih.gov/ij/). We show one representative result. Bars depict the mean ± SD of three independent experiments. ^#^ *p* > 0.05, *p < 0.05, **p < 0.01, and ***p < 0.001 compared with the vehicle (Student’s t-tests).

Hep3B and HepG2 are the two most utilized liver cancer cell lines for *in vitro* toxicity studies (23, 25). We wanted to examine the cell context issue and we treated Hep3B with sodium ascorbate and observed the cytotoxicity at 24 h and 48 h (**Figure 6A**). Sodium ascorbate induced the expression of Nrf2 and HO-1, whereas it downregulated the expression of cyclin D1 and FAS (**Figure 6B**). No endogenous p53 protein was confirmed. In contrast to HepG2, sodium ascorbate elevated the population in the subG1 phase and decreased the populations in the G1 and G2/M phases in Hep3B cells (**Figure 6C**). The inductive or suppressive effect of sodium ascorbate on the S phase was dependent on its concentration. Sodium ascorbate significantly suppressed ROS generation in Hep3B (**Figure 6D**) as did in HepG2 cells (**Figure 2D**). These findings were consistent in that some differences between HepG2 and Hep3B cell lines were described in the literature (24–26).

### The Combination Index of l-AA and TKIs in HepG2 and Hep3B Cells

**Figure 7:**
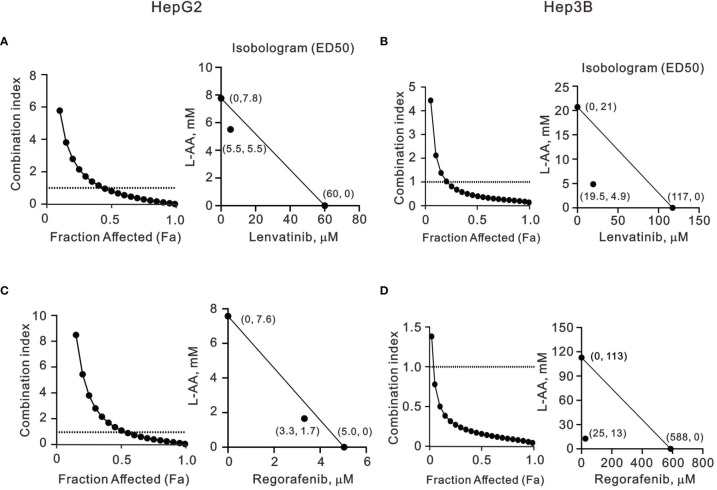
The combination index of TKIs with l-AA in HepG2 and Hep3B cells. **(A, C)** HepG2 and **(B, D)** Hep3B cells were treated with various l-AA doses (0, 0.156, 0.3125, 0.625, 1.25, 2.5, 5, or 10 mM) combined with various lenvatinib doses (0, 0.3125, 0.625, 1.25, 2.5, 5, 10, 20, 40, or 80 μM) or regorafenib doses (0, 0.07813, 0.15625, 0.3125, 0.625, 1.25, 2.5, 5, 10, or 20 μM). Metabolic activity was measured using the MTT method. The combination index of l-AA plus lenvatinib or regorafenib in **(A, C)** HepG2 and **(B, D)** Hep3B cells. Isobolograms (ED_50_) of lenvatinib or regorafenib were calculated using CalcuSyn software.

Our findings revealed that l-AA or sodium ascorbate might play an adjuvant role in the current TKI therapy for HCC patients. Hence, we designed various combinations of concentrations and calculated the combination index (CI) between lenvatinib or regorafenib and l-AA in HepG2 and Hep3B cells as a function of the half-maximal inhibitory concentration (IC_50_) values, the median effective dose (ED_50_) values, and classic experimental design (35). A CI value <1, which denotes a synergistic effect, was observed in most of the tested combinations of lenvatinib or regorafenib and l-AA in HepG2 and Hep3B cells (**Figure 7**). The therapeutic concentration of 60 μM lenvatinib was decreased to 5.5 μM (at 5.5 mM l-AA) in HepG2 cells, while the therapeutic concentration of 117 μM lenvatinib was decreased to 19.5 μM (at 4.9 mM l-AA) in Hep3B cells (**Figures 7A, B**). The therapeutic concentration of 5 μM regorafenib was decreased to 3.3 μM (at 1.7 mM l-AA) in HepG2 cells, while the therapeutic concentration of 588 μM regorafenib was decreased to 25 μM (at 13 mM l-AA) in Hep3B cells (**Figures 7C, D**). These findings suggest the benefit of the clinical application of lenvatinib or regorafenib plus l-AA as a potential adjuvant to overcome the current resistance to VEGFR-targeted TKI therapy in HCC.

## Discussion

In the present study, our TCGA LIHC analysis implied that the ROS-related pathway might correlate with the efficacy of TKI therapy in HCC treatment. Hence, we investigated the cytotoxic effects of pharmacological concentrations of ascorbate (sodium ascorbate or l-AA), which is a well-known redox reagent, alone or combined with TKI therapy on HepG2 and Hep3B cells to figure out the role of ROS in the treatment of HCC. Our study examined the potential functional impact of pharmacological concentrations of ascorbate on the cell-cycle profiles, mitochondrial membrane potential, oxidative response, the synergistic effects of TKIs (lenvatinib and regorafenib), and the differential responsiveness between HepG2 and Hep3B cells. Our study data suggest that the relative level of cell density is an important determinant for ascorbate cytotoxicity in HCC. Our data also revealed that the cytotoxic effect of pharmacological concentrations of ascorbate might not be mediated through our proposed elevation of ROS generation. γH2AX is a well-known biomarker for cellular DNA damage. In our current case, the treatments of either lenvatinib and regorafenib or combined with ascorbate increased the cytotoxicity in a γH2AX-independent manner. Our data uncovered that cellular DNA damage is not the primary cause of cytotoxicity. Other possibilities might lead to these effects, including the induction of DNA repair systems through regulating ROS homeostasis.

There are substantial differences in biochemistry, biology, ethnic origins, and genetics between the HepG2 and Hep3B liver cancer cell lines. HepG2 maintains characteristics more closely related to hepatocytes, whereas Hep3B exhibits more fibroblast-related characteristics and more mesenchymal proteins, indicating an epithelial-to-mesenchymal transition (22, 23, 25). A difference in cell-cycle profiles between HepG2 and Hep3B cells was observed in this study. According to the current literature, instead of a single protein such as p53 or HBx being the crucial factor governing the differences between the HepG2 and Hep3B cell lines, the different origins of biopsy specimens and multiple related factors may be significant. These differences are likely to result in various responses to pharmacological medicines in these two cell lines and lead to opposite outcomes. Other HCCs, such as Huh6 and Huh7 cell lines, and one human normal hepatic cell line Lo2 cell line, were also analyzed in this study, though the effects varied a lot (data not shown). Our combinatory index analysis demonstrated that l-AA combined with lenvatinib and regorafenib expressed synergistic effects in the HepG2 and Hep3B cell lines. Hence, our data suggest that might be a potential candidate for combinatory therapy with VEGFR-targeted TKIs for treating different types of HCC.

A hallmark of HCC is the upregulation of FAS and FAS-related lipogenesis (45). FAS can also affect cell proliferation and anti-apoptotic pathways in a lipogenic-independent manner (46). Our Western blotting analysis demonstrated that sodium ascorbate downregulated the expression of G1 phase, cyclin D1, and FAS proteins, whereas it slightly increased the amount of cleaved PARP in both HepG2 and Hep3B cell lines. However, an increased population in the subG1 was only observed in Hep3B cells. The exact role of FAS in regulating HCC cell proliferation and inhibiting apoptosis needs to be further investigated.

The impact of sorafenib on the oxidative homeostasis of cancer cells occurs through increasing oxidative stress and reducing cellular antioxidant defenses (47, 48). The development of resistance is a primary problem for the antitumor effect of sorafenib or other VEGFR-targeted TKIs in HCC (8, 9). According to our results, ascorbate might be a possible sensitizer for VEGFR-targeted TKIs to overcome the current challenge of clinical resistance. This possibility could further be determined by using TKI-resistant HCC cell lines. In this study, we hypothesized that, since pharmacological concentrations of ascorbate increase ROS production, its combination with VEGFR-targeted TKIs, lenvatinib and regorafenib, would increase intracellular ROS, leading to significant cancer cell death. However, although the combination of ascorbate with lenvatinib and regorafenib exhibited synergistic cytotoxicity in HCC, ROS generation was suppressed by the pharmacological concentrations of ascorbate alone in HepG2 and Hep3B cell lines. Two recent publications demonstrated that pharmacological concentrations of ascorbate might induce or suppress the generation of ROS depending on the type of liver cancer cell lines (49, 50). Compared with the hint from our TCGA LIHC analysis, the ROS issue for the responsiveness of TKI therapy is still puzzling. The efficacy of current combination therapy between TKIs also supports that the downregulation of ROS generation or the upregulation of reductive stress might be involved in the redox homeostasis in HepG2 and Hep3B cells. The potential mechanism remains to be further studied.

The cleavage of histone H3 by sodium ascorbate was observed in HepG2 and Hep3B cells, suggesting other possibilities by ascorbate. Cathepsin L, a lysosomal protease, has been shown to localize in the nucleus, where it participates in the proteolysis of transcription factor CDP/Cux and histone H3 (51). The cleavage site of the histone H3 N-tail targeted by cathepsin L was identified between amino acids 21 and 28, suggesting that N-terminal epigenetic modifications, such as H3K4, H3K9, H3S10, and H3K27, may be disrupted (52). Hence, to better elucidate the cytotoxicity of l-AA in HCC, the involved protease(s) and cleaved site(s) of histone H3 require further investigation.

Recycling of endogenous ascorbate enables continuous production of hydrogen peroxide, which can initiate Fenton’s reactions and cause oxidative damage to cellular macromolecules (53–55). Many studies demonstrated that ascorbate at pharmacologic concentrations might serve as a possible anti-cancer drug acting *via* inducing hydrogen peroxide formation (14–17). However, the hydrogen peroxide production and initiation of Fenton’s reactions through ascorbate oxidation might not be involved in the cytotoxic effect of pharmacological concentrations of ascorbate in HepG2 and Hep3B cells. Hence, the relationships between ascorbate, the formation of hydrogen peroxide and hydroxyl radicals, and the precise equilibria of Fenton’s reaction in HCC remain to be investigated.

## Conclusions

Our findings revealed that the relative level of cell density is an important determinant for the cytotoxicity of HCC due to ascorbate. The cytotoxic effect of pharmacological concentrations of ascorbate might not be mediated through our proposed elevation of ROS generation. Ascorbate might be involved in redox homeostasis to enhance the efficacy of TKIs in HepG2 and Hep3B cells. However, the synergistic effects of ascorbate with TKIs (lenvatinib and regorafenib) support their potential as sensitizers for HCC targeted therapy.

## Data Availability Statement

The original contributions presented in the study are included in the article/supplementary material. Further inquiries can be directed to the corresponding author.

## Author Contributions

Conceptualization, H-LF, Y-LC(hang), S-MH, and T-WC; data curation, H-LF and S-TL; formal analysis, H-LF, S-TL, Y-LC(hang), and Y-LC(hiu); funding acquisition, T-WC; methodology, Y-LC(hang), Y-LC(hiu), and S-MH; project administration, S-MH; supervision, S-MH and T-WC; validation, H-LF and S-TL; writing—original draft, H-LF and T-WC; writing—review and editing, T-WC. All authors read and approved the final manuscript.

## Funding

This work was supported by grants from the Ministry of Science and Technology [MOST–107–2314-B-016-056-MY3 to T-WC] and the Tri-Service General Hospital [TSGH-E-110209 to T-WC], Taiwan, Republic of China.

## Conflict of Interest

The authors declare that the research was conducted in the absence of any commercial or financial relationships that could be construed as a potential conflict of interest.

## Publisher’s Note

All claims expressed in this article are solely those of the authors and do not necessarily represent those of their affiliated organizations, or those of the publisher, the editors and the reviewers. Any product that may be evaluated in this article, or claim that may be made by its manufacturer, is not guaranteed or endorsed by the publisher.

## Abbreviations

ACTN, α-actinin; l-AA, l-ascorbic acid; CI, combination index; CTLA4, cytotoxic T-lymphocyte-associated protein 4; DCFH-DA, 2′,7-dichlorofluorescein diacetate; DMEM, Dulbecco’s modified Eagle’s medium; DsigDB, Drug SIGnatures DataBase; FAS, fatty acid synthase; FACS, fluorescence-activated cell sorting; FBS, fetal bovine serum; FDA, Food and Drug Administration; FSC-H, forward scatter height; GO, Gene Ontology; GSVA, Gene Set Variation Analysis; HCC, hepatocellular carcinoma; HO-1, heme oxygenase-1; ICIs, immune checkpoint inhibitors; LIHC, liver hepatocellular carcinoma; MTT, thiazolyl blue tetrazolium bromide; PARP, poly-ADP-ribose polymerase; PBS, phosphate-buffered saline; PD-1/PDL-1, programmed death-1/programmed death ligand-1; PI, propidium iodide; ROS, reactive oxidative species; SSC-H, side scatter height; TCGA, The Cancer Genome Atlas; TKIs, tyrosine kinase inhibitors; VEGFR, vascular endothelial growth factor receptor.
